# Potential Determinants Contributing to COVID-19 Vaccine Acceptance and Hesitancy in Taiwan: Rapid Qualitative Mixed Methods Study

**DOI:** 10.2196/41364

**Published:** 2023-09-12

**Authors:** Li-Yin Lin, Chun-Ji Lin, Chen-I Kuan, Hung-Yi Chiou

**Affiliations:** 1 Department of Leisure Industry and Health Promotion National Taipei University of Nursing and Health Sciences Taipei Taiwan; 2 Institute of Population Health Sciences National Health Research Institutes Miaoli County Taiwan; 3 Institute of Health Behaviors and Community Sciences College of Public Health National Taiwan University Taipei Taiwan; 4 School of Public Health College of Public Health Taipei Medical University Taipei Taiwan

**Keywords:** COVID-19, vaccine acceptance, vaccine hesitancy, google trends, public health, vaccination, health promotion, thematic analysis, infoveillance

## Abstract

**Background:**

Although vaccination has been shown to be one of the most important interventions, COVID-19 vaccine hesitancy remains one of the top 10 global public health challenges worldwide.

**Objective:**

The objective of this study is to investigate (1) major determinants of vaccine hesitancy, (2) changes in the determinants of vaccine hesitancy at different time periods, and (3) the potential factors affecting vaccine acceptance.

**Methods:**

This study applied a mixed methods approach to explore the potential determinants contributing to vaccine hesitancy among the Taiwanese population. The quantitative design of this study involved using Google Trends search query data. We chose the search term “疫苗“ (vaccine), selected ”台灣” (Taiwan) as the location, and selected the period between December 18, 2020, and July 31, 2021. The rising keywords related to vaccine acceptance and hesitancy were collected. Based on the responses obtained from the qualitative study and the rising keywords obtained in Google Trends, the 3 most popular keywords related to vaccine hesitancy were identified and used as search queries in Google Trends between December 18, 2020, and July 31, 2021, to generate relative search volumes (RSVs). Lastly, autoregressive integrated moving average modeling was used to forecast the RSVs for the 3 keywords between May 29 and July 31, 2021. The estimated RSVs were compared to the observed RSVs in Google Trends within the same time frame.

**Results:**

The 4 prevailing factors responsible for COVID-19 vaccine acceptance and hesitancy were doubts about the government and manufacturers, side effects, deaths associated with vaccination, and efficacy of vaccination. During the vaccine observation period, “political role” was the overarching consideration leading to vaccine hesitancy. During the peak of the pandemic, side effects, death, and vaccine protection were the main factors contributing to vaccine hesitancy. The popularity of the 3 frequently searched keywords “side effects,” “vaccine associated deaths,” and “vaccine protection” continued to rise throughout the pandemic outbreak. Lastly, the highest Google search queries related to COVID-19 vaccines emerged as “side effects” prior to vaccination, deaths associated with vaccines during the period when single vaccines were available, and “side effects” and “vaccine protection” during the period when multiple vaccines were available.

**Conclusions:**

Investigating the key factors influencing COVID-19 vaccine hesitancy appears to be a fundamental task that needs to be undertaken to ensure effective implementation of COVID-19 vaccination. Google Trends may be used as a complementary infoveillance tool by government agencies for future vaccine policy implementation and communication.

## Introduction

COVID-19 is an infectious disease that causes tremendous burden on all health care systems worldwide [1]. Public health authorities are looking for preventive strategies to limit the spread of SARS-CoV-2 because an effective treatment for COVID-19 is not yet to be available [2-4]. According to the Centers for Disease Control and Prevention, to date, vaccines are considered the most powerful therapeutic tools to reduce the spread of infectious viruses such as SARS-CoV-2 [1,5,6]. Although vaccination has been shown to be one of the most important interventions in the field of public health throughout the 21st century, worldwide COVID-19 vaccine hesitancy is one of the top 10 global public health challenges [7,8]. Unlike the development of other previous vaccines, COVID-19 vaccines were exempt from the Food and Drug Administration’s scientific standards and received emergency authorization due to the rapid spread of COVID-19 [9]. Therefore, we proposed that public willingness to receive the COVID-19 vaccine and the keywords selected to perform a web-based search of COVID-19 vaccines may be different from those of other previous vaccines [10-12]. Due to the impact of the global COVID-19 pandemic, the use of traditional data collection methods has been hindered. Recently, many studies have been using Google Trends to collect and analyze data to rapidly capture and reflect health behavior change in different populations [13-16]. Google Trends is a website created by Google LLC, which analyzes the popularity of exact search queries (keywords) on Google across specific regions, time periods, and languages. Recent work has indicated that a mixed methods approach is beneficial for understanding Taiwanese people’s attitudes toward COVID-19 vaccination [15]. The term “mixed methods” refers to the mix of quantitative and qualitative data within a single investigation. This study proposes a mixed methods approach to investigate the following: (1) major determinants of vaccine hesitancy, (2) changes in the determinants of vaccine hesitancy at different time periods, and (3) the potential factors affecting COVID-19 vaccine acceptance among Taiwanese citizens.

## Methods

### Mixed Methods Design

This study used a mixed methods approach to explore the potential determinants contributing to vaccine hesitancy among Taiwanese citizens. The term “mixed methods” refers to collecting and analyzing both quantitative and qualitative data within the same investigation. Mixed methods can draw on potential strengths of both quantitative and qualitative methods, allowing investigators to explore diverse perspectives and discover relationships that exist between layers of multifaceted research questions [17]. The study started with a qualitative analysis to explore the major determinants of vaccine hesitancy and used quantitative analysis to validate the findings by investigating changes in the determinants of vaccine hesitancy at different time periods.

### Qualitative Study

Our study initially involved a qualitative analysis to explore the major determinants of vaccine hesitancy. Web-based interviews were conducted in July 2021, and 31 individual interviews and 1 focus group (comprising 10 participants, a host, and an observer) were completed. We recruited participants through an internet-based bulletin board system as well as from the various social media channels of researchers involved in this study. Although nonrandomized sampling was used in this study, the diversity of the research team members helped to increase the heterogeneity of our sample. In our sample, 15% of participants had less than a junior high school education, 18% of them had a high school education, and 67% of them had a college education or higher. In terms of the level of urbanization of the participants’ areas of residence, 45% of them lived in highly urbanized areas, 33% in moderately urbanized areas, and 22% in lowly urbanized areas. In terms of interview format, the duration of the individual face-to-face interview was approximately 20-40 minutes, while the focus group discussion was organized as a web-based discussion board with a 1-week duration. During that given week, a question would be posted daily on the web-based discussion board, and the participants were encouraged to reply to the question. The responses obtained from the web-based interviews were compiled through thematic analysis to determine the possible determinants contributing to vaccine hesitancy.

### Google Trends Data Collection

**Figure 1:**
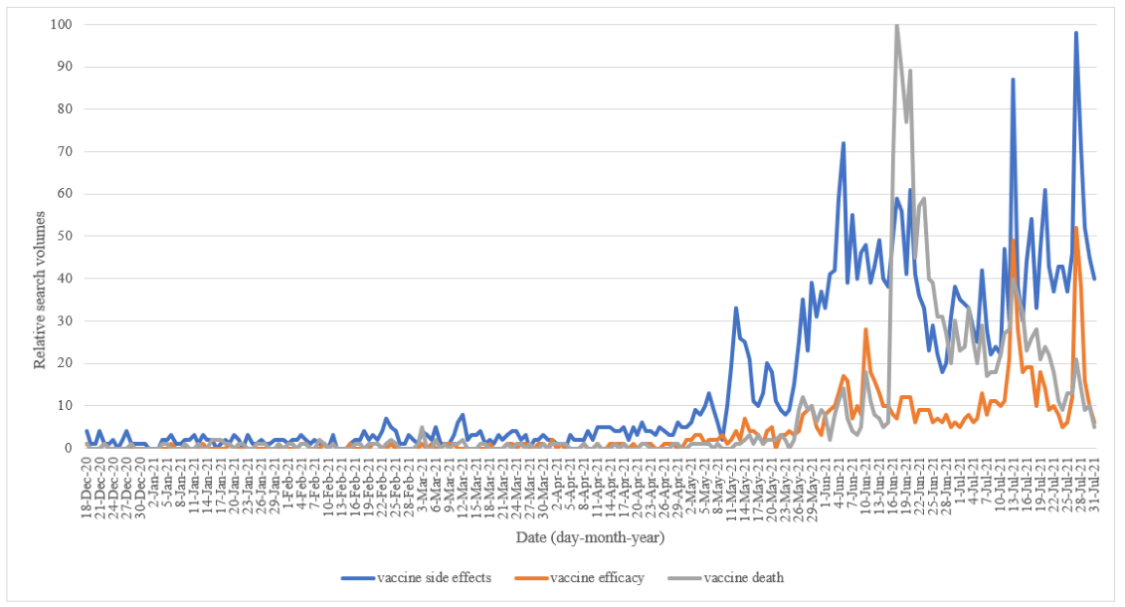
Relative search volumes of COVID-19 vaccine–related web search queries from December 18, 2020, to July 31, 2021, in Taiwan.

The quantitative design of this study involved the use of Google Trends search query data to understand the main factors contributing to vaccine hesitancy. First, we chose the search term “疫苗“ (vaccine), selected ”台灣” (Taiwan) as the location, and selected the period between December 18, 2020, and July 31, 2021, in Google Trends. The search category was set to all categories. We collected the rising keywords related to vaccine acceptance and hesitancy shown in Google Trends (Table 1). Furthermore, based on the responses obtained from our qualitative analysis and the rising keywords obtained in Google Trends, we identified several keywords related to vaccine hesitancy, including “疫苗 副作用” (vaccine side effects), “疫苗 死亡” (vaccine deaths), and “疫苗 保護力” (vaccine protection). Next, these 3 keywords including “疫苗 副作用” (vaccine side effects), “疫苗 死亡” (vaccine deaths), and “疫苗 保護力” (vaccine protection) were used as search queries at the same time in Google Trends between December 18, 2020, and July 31, 2021, to generate relative search volumes (RSVs; Figure 1).

**Table 1:** Rising keywords searched on Google Trends, which are related to vaccine acceptance and hesitancy in Taiwan between May and July 2021.

May 1-31, 2021	June 1-20, 2021	July 1-31, 2021
Government-funded/ vaccine/ appointment	Appointments for leftover doses of vaccines	Vaccine/willingness to get vaccinate/registration
Kaohsiung/ vaccine	Appointments for leftover doses	Vaccine/registration/ system
Vaccine/ order	Vaccine for individuals aged 75 or older	1922 /vaccine/ registration
Medigen/ Vaccine/ Stock shareholder	Sudden death after vaccination	COVID-19 vaccine/ registration and reservation system
Korea/ Vaccine	Vaccination appointments for individuals aged 75 or older	Change of vaccination appointment
Vaccine/ appointment/ Taipei	Vaccination category	1922/ vaccine/appointment/ platform
COVID-19/ vaccine/vaccination	Vaccination appointments of Department of Health, Taipei City Government	Fourth round/ vaccine/ appointment
domestic /vaccine/stocks	Pneumonia/ Pneumococcus/vaccine/where to get vaccination	Medigen/ vaccine/ appointment
Nantou/ vaccine	Pregnant women/Vaccines/ appointments	Second dose/ vaccine/ appointment/ platform
COVID-19/ vaccine/ appointment	Second dose /vaccine/appointment	1922/appointment/get vaccinated
Ko Wen-Je/ vaccine	New Taipei City/vaccine/ appointment system	9th category of qualified population/vaccine/appointment
Vaccine/ insurance package	Guam/ get vaccinated	Vaccine/registration/inquiry
Gou Tai-Ming/ vaccine	vaccine/meaning of unblinding	government-funded /vaccines/ appointment/registration
Vaccine/ allocation	vaccine/ vaccination/ appointment/ platform	1922 /appointments/vaccine
Self-pay/vaccine/eligibility	vaccine/ vaccine leftovers/ registration	1922/ vaccine/ appointment/registration
Taichung/ Hospital/ vaccine	Vaccination appointments of Department of Health, New Taipei City Government	Change/ vaccine/types
Eligibility for vaccination	Vaccine/Get vaccinated/ Classification	1922/vaccine/appointment/system
aged 65 or older/vaccine	Tainan city/vaccines/appointments	Vaccine/ willingness get vaccinated/change
How to make an appointment/get vaccinated	One vial of vaccine can be shared by how many people?	Third round/ vaccine/appointment
Singapore/ vaccine	Vaccination appointments of New Taipei City Government	Modification of vaccination registration
Medigen/ vaccine /chairman	Protein/ subunit/ vaccine	1922/ vaccine/ appointment/inquiry
COVID-19/ vaccine/ side effects	Taichung City/ COVID-19/ vaccine leftovers /vaccine leftovers/ information website	New Taipei city/vaccine/appointment/ platform
AstraZeneca /vaccine/second dose	Get vaccinated/vaccine/death/number of people	Thailand AstraZeneca COVID-19 vaccine
Taiwan/vaccine/quantity	Vaccine/ Good Liver Clinic/vaccine	Government-funded/vaccine/appointment/inquiry
Government-funded/ vaccine/eligibility	Illegal vaccination/vaccine/ list	1992 Vaccine/ get vaccinated/appointments/ platform

### Autoregressive Integrated Moving Average Modeling to Forecast RSVs

We collected the RSV in Google Trends for each search query including “vaccine and side effects,” “vaccine and deaths,” and “vaccines and protection” between December 18, 2020, and July 31, 2021. We applied the autoregressive integrated moving average (ARIMA) modeling algorithm developed by Hyndman and Khandakar [18] to forecast the RSVs of the abovementioned 3 search queries between May 29 and July 31, 2021. In ARIMA modeling analysis, we only used the RSVs of the abovementioned 3 search queries in Google Trends between December 18, 2020, and May 29, 2021, to forecast the RSVs of those 3 search queries between May 29 and July 31, 2021 (assuming that there was no change in the COVID-19 pandemic situation). The forecasted RSVs of those 3 search queries were then compared to their observed RSVs from May 29 to July 31, 2021, in Google Trends.

### Ethical Considerations

This study was approved by the National Taiwan University’s Research Ethics Committee (NTU-Rec No 202106HS026).

## Results

The 4 prevailing factors responsible for COVID-19 vaccines acceptance and hesitancy among Taiwanese citizens were doubts about the government and manufacturers, side effects, deaths associated with vaccination, and efficacy of vaccination. Furthermore, when we combined the qualitative results with the results obtained from Google Trends analysis, “political role” was the overarching consideration leading to vaccine hesitancy during the observed period.

**Figure 2:**
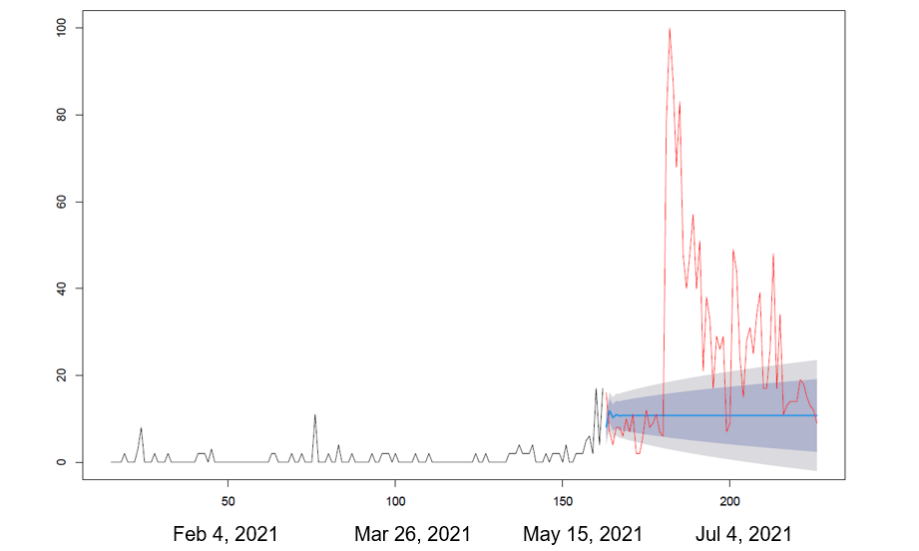
Keywords being searched on Google Trends: “vaccines and deaths.” Statistical analysis was performed using autoregressive integrated moving average modeling. The model was used to observe responses between December 18, 2020 (vaccine production), and May 28, 2021 (vaccination rollout in Taiwan). The time period for comparison was May 29 to July 31, 2021.

**Figure 3:**
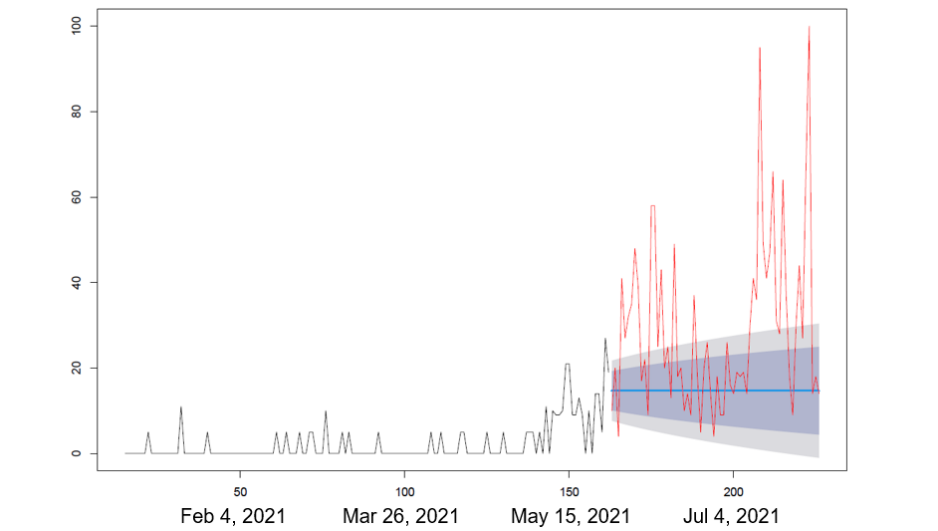
Keywords being searched on Google Trends: “vaccines and side effects.” Statistical analysis was performed using autoregressive integrated moving average modeling. The model was used to observe responses between December 18, 2020 (vaccine production), and May 28, 2021 (vaccination rollout in Taiwan). The time period for comparison was May 29 to July 31, 2021.

**Figure 4:**
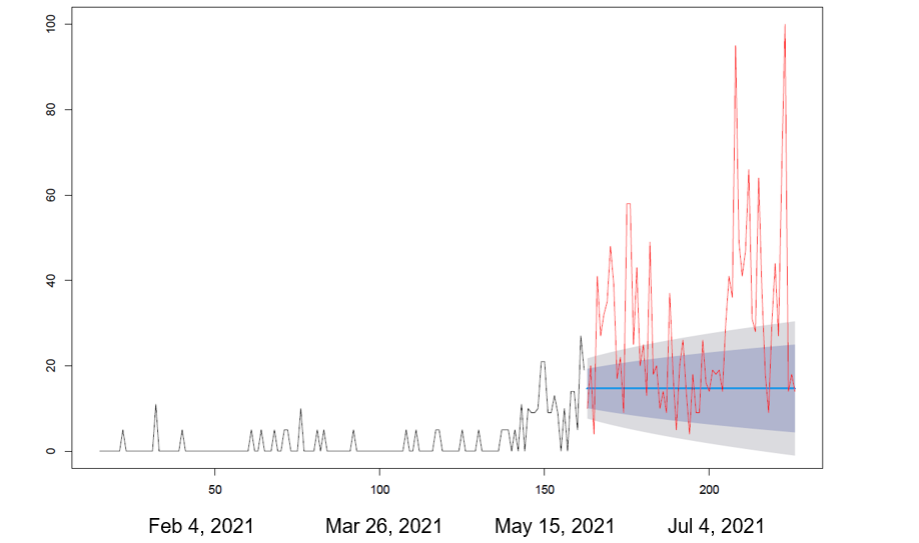
Keywords being searched on Google Trends: “vaccine and protection.” Statistical analysis was performed using autoregressive integrated moving average modeling. The model was used to observe responses between December 18, 2020 (vaccine production), and May 28, 2021 (vaccination rollout in Taiwan). The time period for comparison was May 29 to July 31, 2021.

During the peak of the pandemic, side effects, death, and vaccine efficacy were the main factors contributing to vaccine hesitancy (Table 1). Figure 1 shows the changing trends of factors related to COVID-19 vaccine administration among the Taiwanese population. The first factor of concern before vaccination was the side effects of the vaccine, followed by vaccine-related deaths, and finally the vaccine's protective efficacy. Furthermore, the popularity of the 3 frequently searched keywords, “side effects,” “vaccine associated deaths,” and “vaccine protection,” continued to rise throughout the pandemic outbreak period (Figures 2-4).

Our results show that the RSV of the 3 keywords, “vaccine side effects,” “vaccine deaths,” and “vaccine protection” in Google Trends from May 29, 2021, to July 31, 2021, significantly exceeded the predicted volume based on the RSV before May 29, 2021, from December 18, 2020, to May 28, 2021. In addition, the top Google search queries related to COVID-19 vaccines that emerged were “side effects” prior to vaccination, deaths associated with vaccines during the period when single vaccines were available, and “side effects” and “vaccine protection” during the period when multiple vaccines were available.

## Discussion

### Principal Findings

According to our study, the 4 prevailing factors responsible for COVID-19 vaccine acceptance and hesitancy were doubts about the government and manufacturers, side effects, deaths associated with vaccination, and efficacy of vaccination. During the vaccine observation period, “political role” was the overarching consideration leading to vaccine hesitancy. During the peak of the pandemic, side effects, death, and vaccine efficacy were the main factors contributing to vaccine hesitancy. The popularity of the 3 frequently searched keywords “side effects,” “vaccine associated deaths,” and “vaccine efficacy” continued to rise throughout the pandemic outbreak period.

Public acceptance of a new vaccine is a dynamic phenomenon that is highly influenced by psychological behavior, societal issues, and vaccine-related factors such as safety and efficacy. The introduction and distribution of a new vaccine is indeed an expensive and time-consuming process, while vaccine acceptance is the key indicator that controls the success of vaccination programs [19,20]. Therefore, exploring and investigating the common factors of COVID-19 vaccine hesitancy is a mandatory step in designing an action plan to promote the overall vaccine acceptance rate. Google Trends has often been used by the scientific community to conduct infodemiological and epidemiological analyses [21,22]. This infoveillance approach of studying the distribution and determinants of information via the internet with the intention of informing public health and public policy has been widely applied in various disciplines.

In our analysis, side effects, death, and vaccine efficacy were identified as the main factors contributing to COVID-19 vaccine hesitancy in Taiwan. The extent to which the public trusts the vaccine to be safe and effective after administration appeared to be the strongest predictor of the intention for COVID-19 vaccine uptake. In addition, we also speculated that hidden and miscommunicated health information may accelerate beliefs in antivaccine conspiracies [23]. For instance, misconceptions and mistrust regarding vaccine efficacy were highlighted as the most common reasons to refuse the seasonal influenza vaccine for health care workers in Ireland [24]. A comprehensive review consisting of 2791 studies conducted between 1990 and 2019 showed that although vaccine hesitancy is largely influenced by disease severity, other factors including concerns about vaccine safety and efficacy, culture, and local context are all essential causes of vaccine refusal [25]. To restore trust about vaccines in the public, communication strategies and vaccine delivery processes should be made transparent, accurate, honest, and multimodal and must involve partnerships with community and health care professionals in an inclusive manner.

Similar to many scientific studies in the United States, “political roles” was also identified as a key influencing factor for vaccine acceptance and hesitancy in our analysis. The public does not think that pharmaceutical companies and governments are being transparent in the research findings they release to the public. In this regard, a content analysis of media coverage featuring the COVID-19 issue indicated that politicians were featured as often as, or sometimes even more often than, medical professionals and public health scientists regarding the COVID-19 issue in the United States [26]. In addition, Rzymski et al [27] advocated that evidence-based communication strategies are mandatory to control COVID-19 vaccine–related misinformation in the community and to ensure large public benefits. Therefore, the action that should be taken to reduce vaccine hesitancy would be to communicate effectively using evidence-based information and counteracting messages that can misinform the general public.

### Limitations

There are some drawbacks of using Google Trends data. First, Google Trends data cannot be nationally representative as they overlook responses from other minority groups including those who have low internet access or those with a low socioeconomic status. Second, our study uses several keywords in the Google Search box, rather than a single keyword, during the data collection, which might lead to some potential bias. However, to counteract this drawback, our study design began with a rapid qualitative analysis to serve as the baseline reference for those keywords used in the Google search box in our subsequent quantitative analysis. Lastly, our study does not consider the effect of the media’s influence on the public’s opinions toward vaccines during the pandemic.

### Conclusions

The reluctance toward COVID-19 vaccines continues to be a global concern. In this study, we explored and described the 4 predominant determinants contributing to vaccine hesitancy among Taiwanese citizens. At different pandemic periods, the key influences on the decision to receive a COVID-19 vaccine varied: it began with side effects, followed by deaths associated with vaccination, followed by vaccine efficacy. Furthermore, the political roles have been continuously affecting the Taiwanese population’s attitudes toward vaccination [24,28,29]. Overall, investigating the key factors influencing COVID-19 vaccine hesitancy appears to be a fundamental task that needs to be undertaken to ensure effective implementation of COVID-19 vaccination. This study suggests that Google Trends may be used as a complementary infoveillance tool by government agencies for future vaccine policy implementation and communication.

## Abbreviations

ARIMA: autoregressive integrated moving average
RSV: relative search volume

## Data Availability

The data sets generated during or analyzed in this study are available from the corresponding author on reasonable request.
